# Young-Onset Colon Cancer: A Case Report

**DOI:** 10.7759/cureus.29667

**Published:** 2022-09-27

**Authors:** Syed Salman Hamid Hashmi, Ahmed Shady, Jean Atallah-Vinograd, Donelle Cummings, Ashley Maranino, Jennifer Harley

**Affiliations:** 1 Internal Medicine, NYU (New York University) Langone, Woodhull Medical Center, New York City, USA; 2 Gastroenterology, New York Medical College, Metropolitan Hospital Center, New York City, USA; 3 Advanced Endoscopy/Gastroenterology, New York Medical College, Metropolitan Hospital Center, New York City, USA

**Keywords:** young colon cancer awareness, hispanic health, african american colon cancer, screening colonoscopy, young colon cancer

## Abstract

Colorectal cancer (CRC) which is diagnosed in patients under the age of 50 years is defined as young-onset CRC. There has been a substantial increase in the incidence and mortality of young-onset CRC in the past four decades and the patients have delayed diagnoses leading to the advanced stages of CRC at the time of diagnosis. Here we present a case of a 34-year-old male patient with colon cancer and a literature review on young-onset colon cancer to highlight the age-related disparities in CRC incidence and try to explore the possible causative factors for the rise in incidence and mortality in young patients due to CRC.

## Introduction

Colorectal cancer (CRC) which is diagnosed in patients under the age of 50 years is defined as young-onset CRC [1]. There has been a substantial increase in the incidence and mortality of young-onset CRC in the past four decades and the patients typically have delayed diagnoses due to under-screening of patients under the age of 45 years leading to advanced stages of CRC at the time of diagnosis [1-3]. The annual incidence of young-onset CRC has increased by 2-8% over the past two decades in the United States [1,2]. A total of 21.2% of patients diagnosed with CRC are below the age of 55 and it is a leading cause of death between the age group of 20-49 years [1,2]. Apart from a delay in diagnosis, there may be multifactorial genetic and environmental risk factors that have led to an increased incidence of CRC [3-5]. We present a case of a 34-year-old male patient with colon cancer who initially presented to the hospital with anemia. We illustrated the age-related disparities in CRC incidence and tried to explore the possible causative factors for the rise in incidence and mortality in young patients due to CRC through a literature review. A literature review was conducted using the PubMed database that included prospective trials, meta-analyses, and systematic reviews for the last 10 years from January 2012 through March 18, 2022; a total of 40 titles underwent full-length article assessment.

## Case presentation

**Figure 1:**
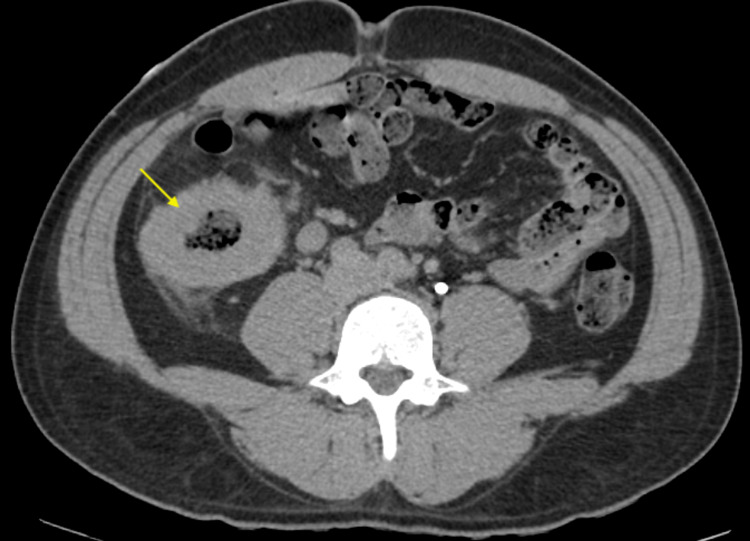
CT abdomen and pelvis with contrast shows a circumferential wall thickening of the proximal ascending colon with prominent lymph nodes (arrow).

A 34-year-old Ecuadorian male with no past medical or surgical history was referred by his primary care physician for low hemoglobin of 3.1 g/dL. The patient did not have a history of gastrointestinal bleeding, alcohol consumption, or smoking. The patient immigrated to the United States from Ecuador one year prior to his presentation and complained of fatigue, headache, lightheadedness, and early satiety for eight months. The patient was admitted to the hospital for symptomatic anemia evaluation and he received three units of blood transfusion after which his hemoglobin normalized. There was no family history of gastrointestinal malignancies. Anemia workup revealed iron deficiency anemia. A CT abdomen and the pelvis showed a 9 cm circumferential wall thickening of the proximal ascending colon with prominent lymph nodes in the abdomen (Figure 1). CT chest and a CT triple phase of the liver ruled out metastasis.

**Figure 2:**
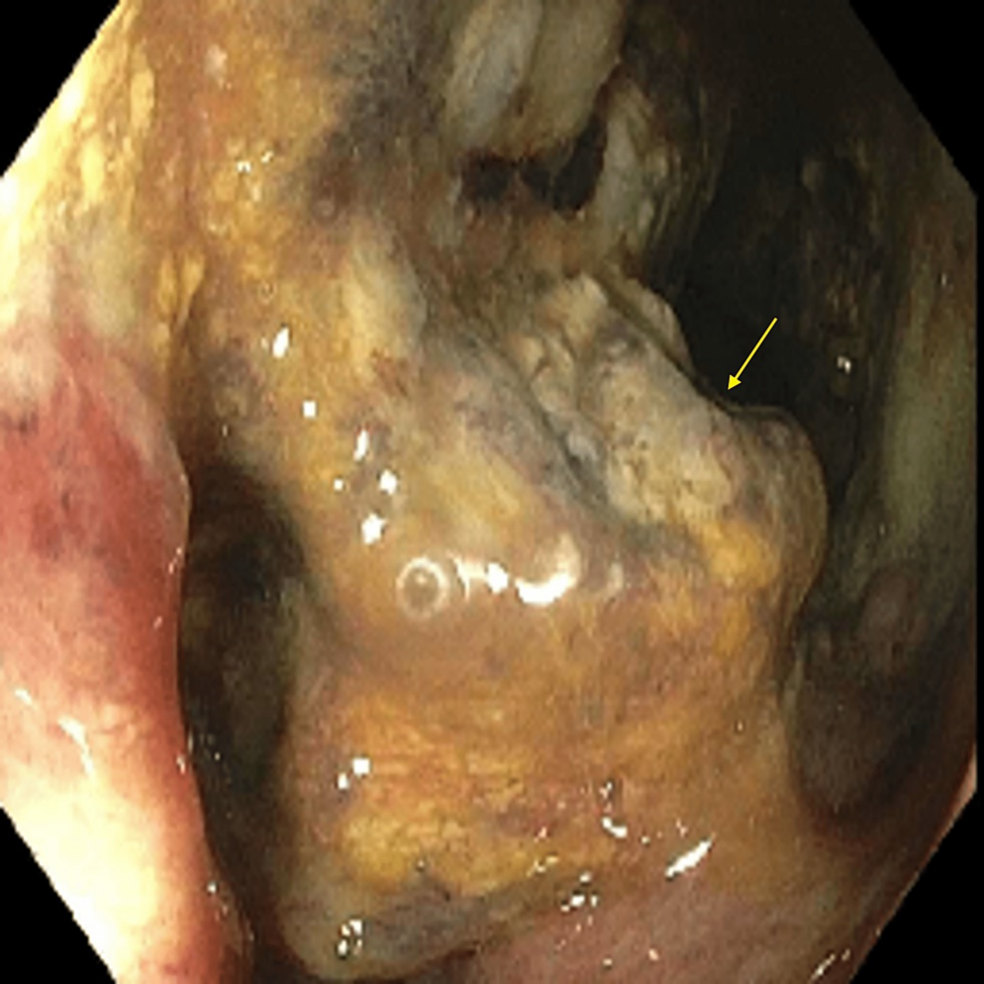
Colonoscopy image shows a 30 mm ulcerated, circumferential mass extending from the ileocecal valve to the hepatic flexure. The arrow shows the mass partially blocking the lumen.

**Figure 3:**
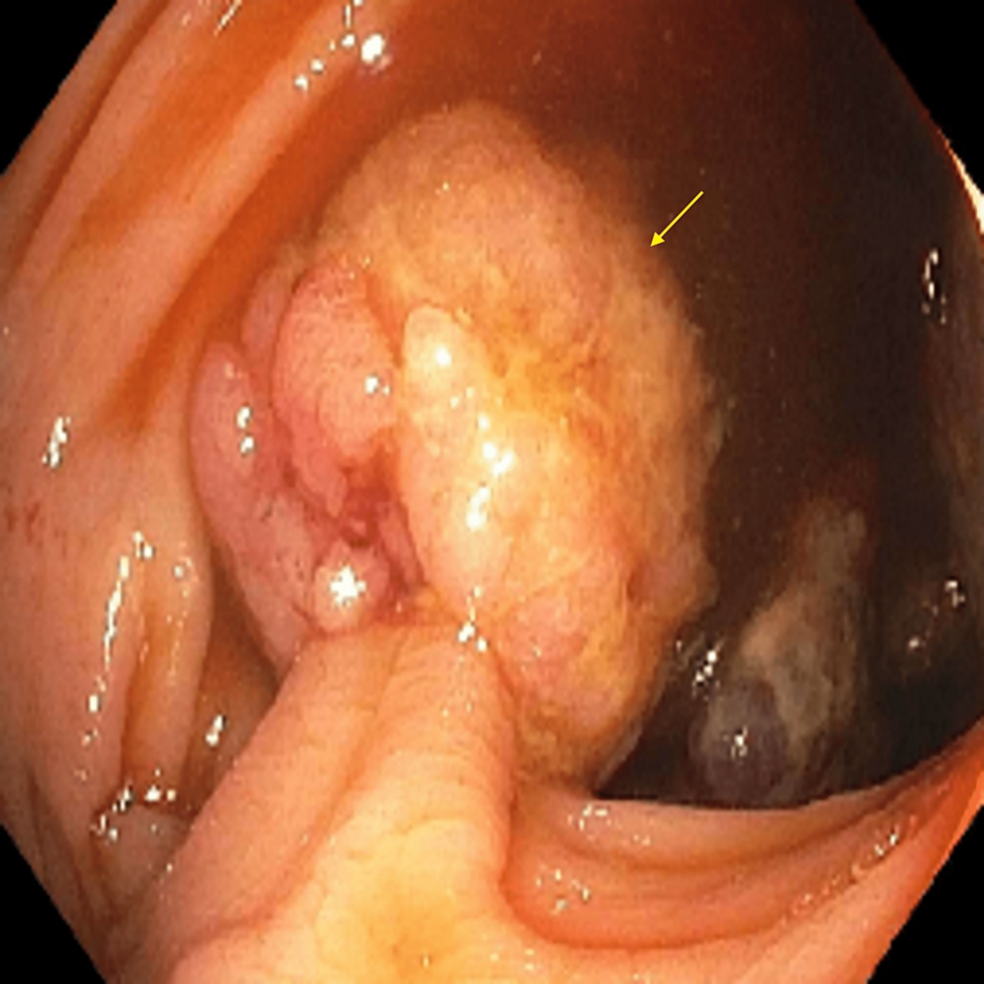
Colonoscopy image shows a 20 mm polyp (arrow) at the hepatic flexure.

**Figure 4:**
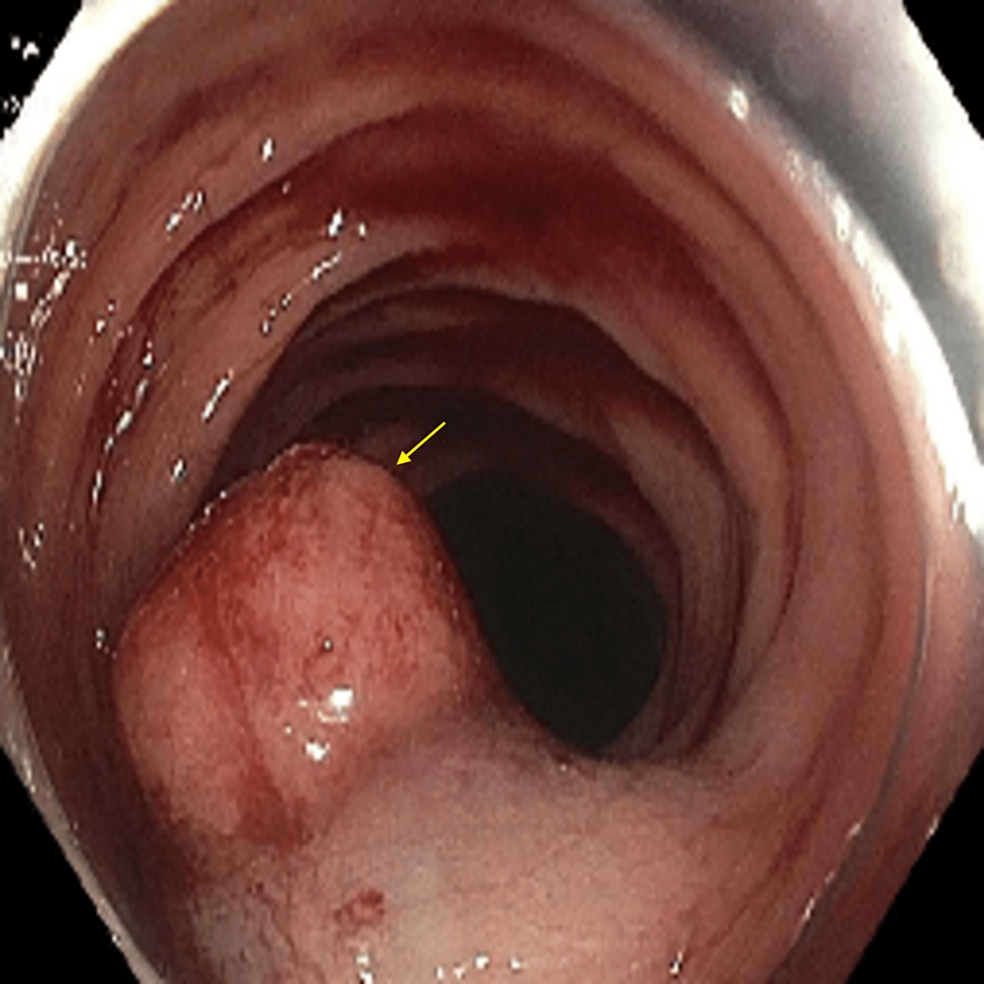
Colonoscopy image shows a 20 mm polyp (arrow) in the descending colon.

**Figure 5:**
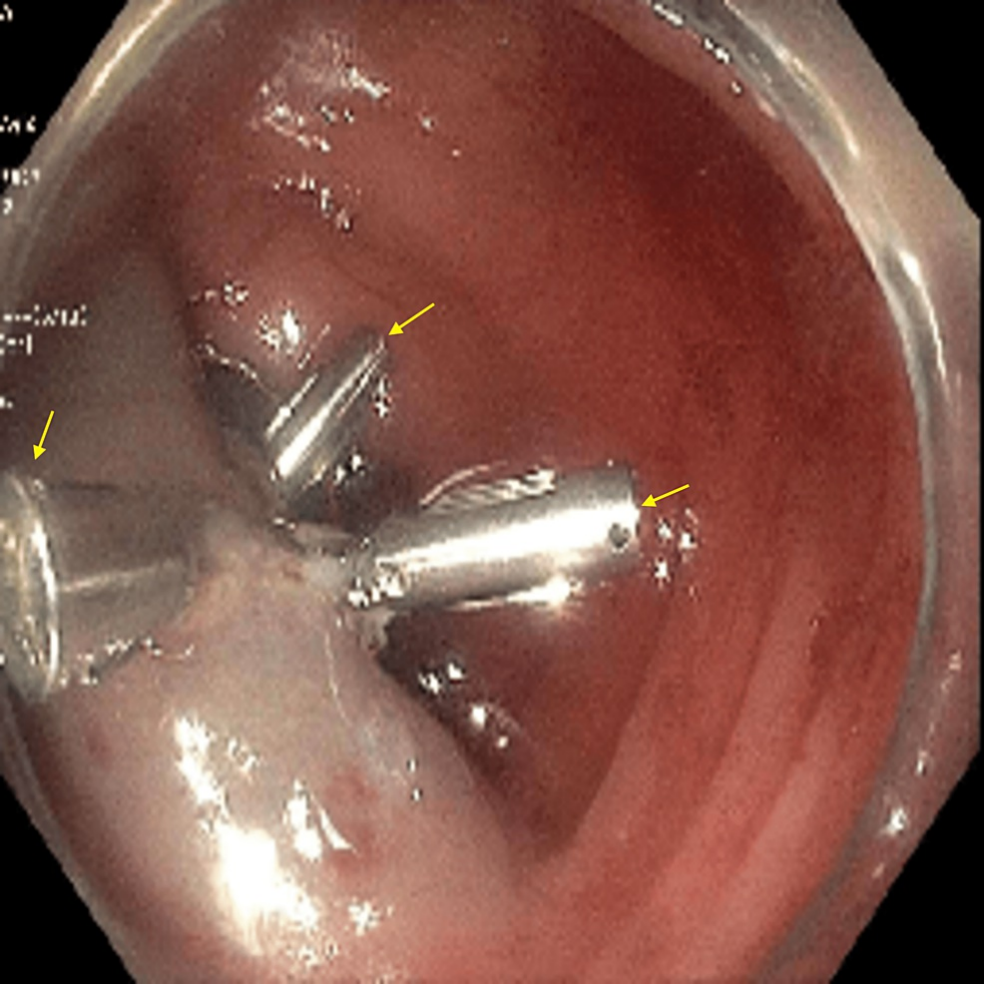
Colonoscopy image shows the descending colon polyp removal by endoscopic mucosal resection. The arrows show the endoclips which were applied for hemostasis.

Carcinoembryonic antigen (CEA) level was 6.1 ng/mL (reference range: 0-2.5 ng/mL). The patient received a colonoscopy which revealed a 30 mm ulcerated, circumferential mass extending from the ileocecal valve to the hepatic flexure (Figure 2). The colonoscopy also revealed a 20 mm polyp which was found at the hepatic flexure that was removed by polypectomy (Figure 3), and a 20 mm polyp in the descending colon (Figure 4) that was removed by endoscopic mucosal resection (EMR) (Figure 5).

Histopathology of the circumferential mass showed invasive poorly differentiated adenocarcinoma and the histopathology of the hepatic flexure polyp, and descending colon polyp showed tubulovillous adenoma and tubular adenoma. The patient underwent a right hemicolectomy and was started on chemotherapy as recommended by oncology. CEA levels normalized six months post-chemotherapy and a CT abdomen and pelvis showed no metastasis.

## Discussion

Routine screening colonoscopies have considerably decreased the overall incidence and mortality of CRC in the last few decades. But for patients under the age of 50 years, the incidence rate of CRC has increased in the past three decades [1,6]. The Surveillance, Epidemiology, and End Results (SEER) data from 1984 to 2020 showed that in the United States among those under the age of 50 years, there was a 2.6% annual increase in the incidence rate of CRC, with the greatest increase in CRC incidence rates seen among patients between ages of 20 and 34 years [1,7]. In the United States, young-onset CRC is the second leading cause of cancer among males and the fourth leading cause of cancer among females [1,8]. There are substantial racial and ethnic differences in CRC incidence in the United States [1,9]. The New York Cancer Registry showed an incidence of 29% of South Asians and 26% of Southeast Asians were under age 50 at diagnosis, compared to 14% of non-Hispanic whites. Non-Hispanic Black Americans are disproportionately diagnosed with a greater prevalence of CRC at later stages of the disease and have poorer overall survival rates [10-12]. A family history of colon cancer is the strongest known risk factor for CRC and close to 23-39% of young-onset CRC patients have a family history of CRC [13-16]. Inflammatory bowel disease (IBD) is also a risk factor for CRC and is associated with mucinous or signet ring histology [15].

CRC is often asymptomatic but may present with red-flag symptoms such as unexplained anemia, and rectal bleeding. Young-onset CRC patients predominantly present with hematochezia, change in bowel habits, abdominal pain, anemia, and weight loss [1,17-24]. Precursor adenomatous lesions are less likely to have associated young-onset CRCs [24]. They generally occur distal to the splenic flexure or the rectum [25,26]. Literature review shows a seven-week to a two-year delay in diagnosis of young-onset colon cancer probably due to a low level of suspicion for CRC when the patient presents with symptoms. Young-onset CRC also tends to be diagnosed at later stages with metastatic disease. The increase in young-onset CRC is attributed to behavioral, lifestyle, and environmental factors that influence disease risk [1,18,19,9-16]. Heavy alcohol consumption, obesity, diabetes, and lack of physical activity are associated with an increased risk of CRC [27-42]. The American Cancer Society (ACS) and US Preventive Services Task Force (USPSTF) recently modified the guidelines for screening colonoscopies to include the age between 45 and 49 years. These recommendations for screening below the age of 50 years are based on modeling studies evaluating young adults receiving colonoscopies because of symptoms and family history. Aggressive histologic characteristics are frequently seen in young-onset CRC [37].

## Conclusions

The case report and the literature review highlight the importance of being vigilant of CRC symptoms even in the young population, especially when they have risk factors for CRC. The article also emphasizes the age-related and racial disparity in CRC distribution. It also identifies a need to increase the awareness of young-onset CRC among clinicians and patients, especially in the minority communities like the African American and Hispanic communities.
